# Temporal and Spatial Patterns of Mating in *Rhodnius prolixus*

**DOI:** 10.3390/insects16030312

**Published:** 2025-03-18

**Authors:** Franco Divito, Gabriel A. De Simone, Lorena Pompilio, Gabriel Manrique

**Affiliations:** 1 Laboratorio de Fisiología de Insectos, Departamento de Biodiversidad y Biología Experimental, Facultad de Ciencias Exactas y Naturales, Universidad de Buenos Aires, IBBEA, UBA-CONICET, Buenos Aires C1428EGA, Argentina; francodivito92@gmail.com (F.D.); gdesimone@outlook.com (G.A.D.S.); 2 Laboratorio de Ecología y Comportamiento Animal, Departamento de Ecología, Genética y Evolución, Facultad de Ciencias Exactas y Naturales, Universidad de Buenos Aires, IEGEBA, UBA-CONICET, Buenos Aires C1428EGA, Argentina; lopompilio@yahoo.com.ar

**Keywords:** circadian rhythms, endogenous control, sexual behavior, shelters, triatomines

## Abstract

The kissing bug *Rhodnius prolixus* is a nocturnal species; however, its temporal mating patterns remain unexplored. Copulation lasts about fifty minutes, during which time the couple remains almost motionless. We hypothesized that *R. prolixus* copulates within shelters to reduce its vulnerability. To test this, we examined the spatial and temporal patterns of its copulation, as well as the potential endogenous regulation of its circadian rhythm, under three conditions: a light:dark cycle (L/D), constant light (L/L), and constant darkness (D/D). Over ten days, the number and the timing of copulation of pairs in arenas containing a shelter were video-recorded. Under the L/D cycle, the pairs mated mainly during daylight hours. This rhythm persisted under the D/D cycle, with a peak extending from the first half of the subjective day to the first half of the subjective night. No rhythm was observed under the L/L cycle. A greater proportion of mating occurred within the shelters during the L/D and L/L cycles, whereas its spatial distribution was random under the D/D cycle. Our results reveal an endogenously controlled circadian rhythm of mating behavior, with mating activity mainly occurring during daylight hours. Additionally, we showed that the pairs copulated inside the shelters. These results provide new insights into the reproductive behavior of *R. prolixus*.

## Simple Summary

This study examined the existence of a circadian mating rhythm and the use of shelters during copulation in the kissing bug *Rhodnius prolixus*. For this purpose, the mating activity of single pairs was registered for ten consecutive days under three light:dark conditions: a normal light:dark cycle, constant light, and constant darkness. The results show that *R. prolixus* mates mainly during the light phase of the light:dark cycle. This rhythm persists under constant darkness but not under constant light, suggesting endogenous control. Mating predominantly occurred inside the shelters under the light:dark cycle and constant light, whereas this distribution was random under constant darkness. These findings suggest that despite its primarily nocturnal habits, *R. prolixus* copulates during the day when protected within shelters.

## 1. Introduction

The day–night cycle generates drastic changes in the environment, affecting the lives of most organisms. To withstand these changes better, animals have developed rhythmic patterns in their physiology, metabolism, and behavior, which vary according to the hour of the day and play key roles in their survival. Animals have “clock” genes that enable them to anticipate daily changes and contribute to the maintenance of rhythms, even in the absence of external inputs [1,2]. A biological rhythm that occurs in an approximately 24 h period and is reset by environmental cues (such as light or temperature) is called a circadian rhythm [2,3]. Insects have been extensively used in chronobiological studies [4,5,6,7]. Among these arthropods, the rhythms of those that are disease vectors have been widely studied, not only because of their public health importance but also because they provide valuable insights into the adaptive value of temporal organization [8,9]. As vectors live at the expense of hosts, the timing of their activities is not only modulated by the environmental conditions and the avoidance of predators and parasites but is also heavily influenced by the hosts’ own biological rhythms. For example, behaviors related to feeding, such as host-seeking, usually coincide with the times at which hosts are less active [8,9].

*Rhodnius prolixus* (Hemiptera: Reduviidae: Triatominae) plays a significant role as a vector in the transmission of Chagas disease in the Americas, along with many other species within the same subfamily [10]. Some biological rhythms have been studied in *R. prolixus* and other related triatomine species. These hematophagous insects have gregarious and mainly nocturnal habits, actively searching for food at night and remaining aggregated in an akinetic state inside shelter during the day. Previous studies in *Triatoma infestans*, *R. prolixus*, and *R. robustus* have identified a bimodal pattern of locomotor activity in these species, characterized by a primary peak at dusk and a secondary peak at dawn. Their activity displayed at dusk has been associated with host-seeking, while their activity at dawn has been linked with the search for shelter to return to after feeding [11,12,13]. In accordance with these patterns, both *R. prolixus* and *T. infestans* show higher responsiveness to CO_2_ (which serves as a cue for the presence of hosts) at dusk and to aggregation pheromones at dawn [14,15,16]. Additionally, *T. infestans* is more sensitive to light (negative phototaxis) during the scotophase, which is part of a defensive strategy to avoid detection during periods of heightened activity [17]. A rhythmic pattern of thermopreference has also been reported in triatomines. The preferred temperature in *T. infestans* and *R. prolixus* is higher at the beginning of the scotophase and lower at the beginning of the photophase [18,19]. Since the metabolic rate increases with rising temperatures in insects, these results may be interpreted as an adaptation in triatomines to save energy when their blood storage is maximal and to process food only when necessary. Additionally, the proboscis extension response (PER) to heat (which is crucial for feeding) is triggered in *R. prolixus* more frequently during the initial hours of night [20]. These studies have contributed to formulate hypotheses regarding many aspects of the behavior of triatomines in nature. However, one important physiological process whose rhythmicity remains unexplored in triatomines is mating. This is particularly notable given that mating rhythms have been studied in other insects, such as other hemipterans, beetles, and cockroaches [21,22,23,24].

*Rhodnius prolixus* is a widely utilized model organism for the investigation of insect reproduction and physiology. Briefly, successful mating in this species begins with the male jumping onto or mounting the female. Subsequently, the male orientates himself in the same direction as the female and places himself in her lateral region, grasping her with his three pairs of legs. Once the male has secured his position, contact between the genitalia of both individuals occurs, thereby initiating copulation [25,26,27]. The duration of copulation in *R. prolixus* is approximately fifty minutes, during which time the insects remain almost completely stationary [25,26]. Although the physiological and behavioral patterns of *R. prolixus* mating have been extensively studied, it is still unknown whether this species exhibits a biological rhythm in mating and what its specific characteristics are [26,28,29]. Previous works have provided some indirect evidence regarding the timing of sexual activity in *R. prolixus*. During the initial hours of the night, females show a tendency to release compounds from their metasternal glands which are linked to sexual communication [30]. Additionally, males show a tendency to remain outside their shelters during the night if female odors are present in the environment [31]. The high level of locomotor activity exhibited by triatomines near dusk may also suggest that these insects not only search for hosts when they become active but also for potential mates. However, the temporal pattern of mating itself has not yet been investigated, which will be one of the objectives of this study.

As previously mentioned, triatomines spend the daylight hours in shelter, which provides protection from predators and other hazards in their habitats, and emerge at night to feed [32]. It is also known that *R. prolixus* exhibits a higher locomotor activity and exits its shelters more frequently when a host is present in the surrounding environment [33]. As a consequence of their strong thigmotaxis and negative phototaxis, triatomines mostly utilize dark and narrow locations with irregular surfaces as shelters during periods of low activity. *Rhodnius* spp. are very capable of colonizing human dwellings, where they have plenty of places to hide, and sylvatic populations are commonly associated with palm trees [34]. Triatomines actively choose their shelters based on a range of environmental factors, including temperature, relative humidity, light conditions, height, and shelter position (vertical/horizontal) [35,36,37]. Zacharias et al. [36] also found that shelter usage depends on the density of individuals and that differences in the rates of shelter occupancy during the photophase also depend on developmental stage, nutritional status, and order of arrival. Additionally, the competitive ability to occupy shelter varies between species, as *Triatoma* spp. are more efficient in this process than *R. prolixus* [36]. One aspect that remains unknown is the spatial pattern of mating, i.e., whether it occurs inside or outside shelter. Lazzari et al. [32] have stated that these places not only have protective functions but may also facilitate intraspecific interactions, including searching for a mate. However, no work has yet confirmed that triatomines use shelters for this purpose.

This study aims to describe the temporal and spatial patterns of mating in *R. prolixus*. Experiments were conducted using single pairs released into an experimental arena containing a shelter under three photoperiod conditions: a 12 h:12 h light:dark illumination regime, constant light, and constant dark. To investigate the temporal pattern, we recorded the number of matings of pairs of *R. prolixus* placed in the experimental arena over ten consecutive days and registered the timing of each mating event. A light:dark cycle was used to determine whether *R. prolixus* exhibited a mating rhythm under normal day–night conditions. Constant photoperiods (in free-running conditions) were employed to assess whether a mating rhythm persisted in the absence of external inputs, indicating endogenous control. To study the spatial pattern, we determined whether mating in *R. prolixus* occurred inside or outside the artificial shelters. We hypothesized that *R. prolixus* mating shows a rhythmic pattern that matched with the hours of higher activity reported for this species and took place in locations offering greater protection. Thus, we predicted that *R. prolixus* mating would present a circadian rhythm with a peak during night and that this rhythm would be under endogenous control. We also predicted that a greater proportion of mating would occur inside the shelter, regardless of the light:dark cycle.

Based on the results obtained, we analyze the relationship between the observed mating rhythm and the other behavioral rhythms reported for triatomines. Additionally, we discuss its potential contribution to the survival and ecological adaptations of *R. prolixus*’s reproductive biology.

## 2. Materials and Methods

### 2.1. Insects

The *R. prolixus* specimens used in this study were reared in our laboratory insectary from eggs under controlled conditions: a temperature of 28 ± 2 °C, a relative humidity of 30–60%, and a 12 h:12 h light:dark illumination regime (lights on at 20:00, lights off at 8:00). All nymphal instars were fed weekly ad libitum for about 45 min on live hens. During feeding, the insects were kept inside cylindrical acrylic flasks (9.5 cm high × 8.5 cm diameter) with a piece of filter paper as the substrate and a nylon mesh as a cover and a floor. Each flask was gently placed in close contact with the hen’s skin, and therefore the insects used the substrate to walk and reach the food source. Insects were sorted by sex immediately after the imaginal ecdysis.

Virgin adults were segregated into different acrylic flasks (9.5 cm high × 8.5 cm diameter) until the experiments were performed. The males and females used in this study were 31 days old, fed four times on a weekly basis starting from day 7 post-ecdysis to ensure that they fed to repletion. When virgin *R. prolixus* males and females are well fed, they are prone to copulate [38]. The bugs were not fed for three days prior to the start of the experiments and were entrained during this period in the 12 h:12 h light:dark illumination regime (lights on at 8:00, lights off at 20:00).

### 2.2. The Experimental Device

**Figure 1:**
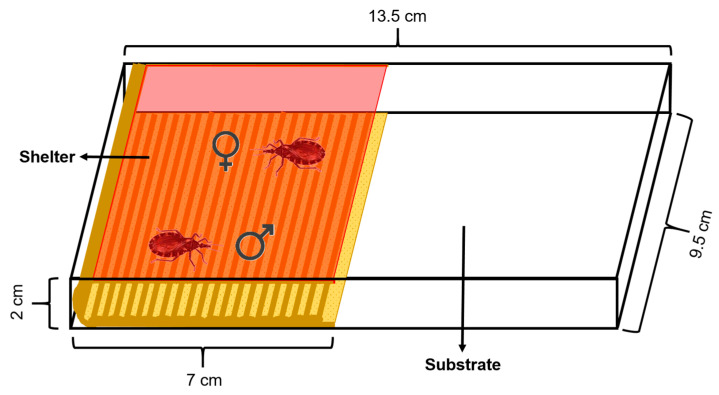
Diagram of the experimental arena. The shelter, which consists of a corrugated cardboard floor and a red acetate ceiling, occupies one half of the arena. A male and female *R. prolixus* (shown inside the shelter) are enclosed inside the arena and can interact freely. A removable lid covers the arena and prevents the insects escaping.

The experimental arena used consisted of a rectangular acrylic box (13.5 cm length × 9.5 cm width × 2 cm height) covered by a removable lid. The floor was covered with a piece of filter paper as the substrate which was changed between assays. An artificial shelter was placed on the arena over the substrate. This shelter (7 cm length × 9.5 cm width × 2 cm height) consisted of a piece of corrugated cardboard as the floor and a piece of red transparent acetate attached to it that functioned as a ceiling, allowing mating inside the shelter to be video-recorded (Figure 1). *Rhodnius prolixus* cannot perceive red light, so the shelter was assumed to be mostly dark for these insects [39]. The area of the arena had two equal halves, one occupied with the shelter and the other one by the substrate. The shelter was accessible to the insects only from the front but not from the sides, minimizing the entrance of light. Additionally, the lid covering the shelter prevented the insects from walking over it.

The behavior of the insects was registered under three different photoperiods (see Section 2.3). For the light periods, two laterally placed lamps generated a homogeneous illumination intensity of 35 lux (digital luxometer TES-1330; Tes Electrical Electronic Corp., Taipei, Taiwan) over the arena. During the dark periods, complete darkness was set.

### 2.3. The Procedure

The manipulated variable was the photoperiod. Three photoperiod regimes were randomly assigned to each couple: (a) a light:dark regime, identical to that used during training (lights on at 8:00, lights off at 20:00) (L/D); (b) a constant dark regime (D/D); and (c) a constant light regime (L/L).

For each photoperiod, 24 replicates were conducted. In each replicate, one male and one female *R. prolixus* were released into the arena. To avoid mechanical disturbance triggering discharge from the Brindley’s and/or metasternal glands, each individual was allowed to climb onto a piece of filter paper, which was then placed in contact with the surface of the arena, letting the insects walk freely when they were released [40]. Once the insects were placed in the arena, it was immediately covered with the lid, thereby starting the measurements. The insects could then interact freely for ten consecutive days. All experiments were performed in a closed experimental room with controlled temperature (26.0 ± 0.5 °C) and relative humidity (30–60%). The behavior of the insects was recorded using web cameras (Olex.la, K7004D, China) placed 40 cm above the arena. This procedure allowed the experimenter to observe the matings without disturbing the bugs. As the cameras had infrared lights, we could monitor the behavior of the insects even in complete darkness.

To study the temporal pattern of mating, we established four time intervals of 6 h each: 8:00–14:00 (the early photophase for L/D or early in the subjective day for constant conditions), 14:00–20:00 (the late photophase for L/D or late in the subjective day for constant conditions), 20:00–2:00 (the early scotophase for L/D or early in the subjective night for constant conditions), and 2:00–8:00 (the late scotophase for L/D or late in the subjective night for constant conditions) (GMT-3). The following variables were registered for each replicate, reproducing the video recordings: (1) the total number of matings; (2) the number of matings that occurred at every time interval (registering the date and time of each mating); and (3) the proportion of matings inside the shelters to the total mating.

In order to minimize the impact of the starting time on the probable mating rhythm, sets of 8 assays each were started at different times for each photoperiod: 14:00, 16:00, and 18:00 (GMT-3). When the assays ended, the insects were sent back to the insectary. Any eggs or first-instar nymphs resulting from the matings were sent to the insectary as well.

### 2.4. The Data Analysis

The temporal pattern of mating of *R. prolixus* was analyzed using a generalized linear mixed model (GLMM) with a repeated measures design (RMD) and a Poisson distribution. The photoperiod (L/D, D/D, and L/L) and the time interval (8:00–14:00, 14:00–20:00, 20:00–2:00, and 2:00–8:00) were used as the explanatory variables, and the registered number of matings at every time interval in each photoperiod was the dependent variable. Post hoc Tukey’s tests were conducted in case of a significant interaction between the main factors.

To assess the spatial pattern of mating, we compared the proportion of mating inside the shelter to the total mating using Student’s *t*-tests. We performed one analysis for each photoperiod (L/D, D/D, and L/L). As the shelter comprised half of the surface of the arena, we expected a proportion of 50% of mating inside the shelter as the null hypothesis in each photoperiod. Additionally, we analyzed differences in the proportion of mating inside the shelter between photoperiods using a generalized linear model (GLM) with a link logit and a binomial distribution. In this analysis, the photoperiod (L/D, D/D, and L/L) was the explanatory variable, and the proportion of mating inside the shelter to the total mating was the dependent variable. Post hoc Tukey’s tests were conducted when this analysis found significant differences to determine which levels of the photoperiod treatment were different from each other.

Additionally, as controls, we aimed to confirm that differences in the light:dark cycle did not affect the sexual activity displayed by *R. prolixus* nor the days on which mating took place. To this end, we performed two separate analyses. In the first one, we compared differences in the sexual activity between photoperiods (measured as the total number of matings per couple during the ten experimental days). For this analysis, we performed a GLM with a Conway-Maxwell-Poisson distribution. The photoperiod (L/D, D/D, and L/L) was the explanatory variable, and the number of matings per pair was the dependent variable. For the second, we analyzed the effects of the photoperiod on the distribution of mating across days, examining the days on which mating occurred and comparing its distribution among the treatments. This study was necessary because the experiment spanned several days, during which time the bugs did not feed and only interacted with their partner in the arena, so it was expected that the sexual activity of the couples would decrease at some point. This decrease in sexual motivation could result from an increasing time without feeding [41] and/or a lack of novel partners, as observed in other insects [42]. For this analysis, we performed a GLMM with a random effect structure and a Conway-Maxwell-Poisson distribution. The photoperiod (L/D, D/D, and L/L) and the day interval (1–2, 3–4, 5–6, 7–8, and 9–10) were used as explanatory variables, and the number of matings at each day interval in each photoperiod was the dependent variable. Post hoc Tukey’s tests were conducted when this analysis found significant differences to determine which levels of the photoperiod treatment were different from each other.

All of the statistical analyses were performed using R Statistical Software (v4.2.0; R Core Team, 2022).

## 3. Results

At least one mating was observed in each assay; thus, no assays were excluded from the analyses. Additionally, no matings were observed beyond the ninth day of the assays.

### 3.1. The Temporal Pattern of Mating in R. prolixus

**Figure 2:**
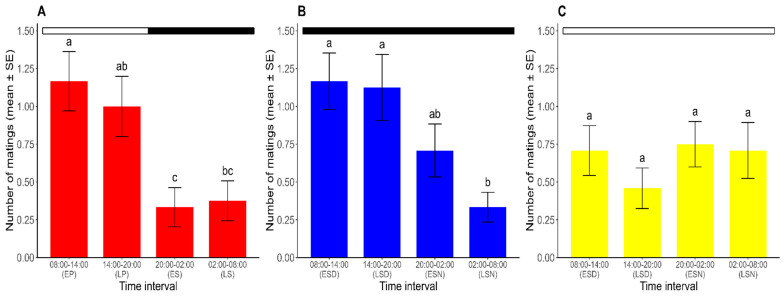
Number of matings for each time interval over ten days (mean ± SE) under (**A**) L/D, (**B**) D/D, and (**C**) L/L regimes. For each photoperiod, different lowercase letters represent significant differences between time intervals (*p* < 0.05). Dark and white horizontal bars indicate the periods of the lights being off and on, respectively. EP: early photophase. LP: late photophase. ES: early scotophase. LS: late scotophase. ESD: early subjective day. LSD: late subjective day. ESN: early subjective night. LSN: late subjective night. (*n* = 24 for each photoperiod).

Mating occurred throughout the 24 h period in all photoperiods. For the analysis of the temporal pattern of mating in *R. prolixus*, the results of the GLMM with a RMD, with the photoperiod (L/D, D/D, and L/L) and the time interval (8:00–14:00, 14:00–20:00, 20:00–2:00, and 2:00–8:00) as explanatory variables and the number of matings as the dependent variable, showed significant differences in the number of matings across time intervals (χ^2^ = 14.531, df = 3, *p* < 0.05). The analysis also revealed a significant interaction between the two factors (χ^2^ = 15.466, df = 6, *p* < 0.05). A post hoc analysis compared the effect of the time interval on the number of matings for each photoperiod. Thus, under the L/D regime, the results revealed a peak in the mating activity during the photophase (8:00–20:00), demonstrating the existence of a mating rhythm in *R. prolixus*. Equally, the mating activity was higher in the early photophase. The number of matings during this interval did not significantly differ from that at the late photophase (Tukey’s test; *p* > 0.05), but it was significantly higher than the numbers registered during both the early and late scotophase (Tukey’s test; *p* < 0.05). The mating activity during the late photophase was significantly higher than that observed during the early scotophase (Tukey’s test; *p* < 0.05) but did not differ significantly from the late scotophase (Tukey’s test; *p* > 0.05). Furthermore, no significant differences were found in the number of matings recorded between the two halves of the scotophase (Tukey’s test; *p* > 0.05) (Figure 2A).

Under constant darkness (the D/D regime), the mating rhythm persisted, although it extended from 8:00 to 2:00. A higher number of matings was found during the early subjective day, which did not differ significantly from the numbers of matings in the late subjective day and early subjective night (Tukey’s test; *p* > 0.05). The number of matings during the late subjective night, when the sexual activity was at its lowest point, significantly differed from that found in each half of the subjective day (Tukey’s test; *p* > 0.05) but did not differ from the number of matings observed in the early subjective night (Tukey’s test; *p* < 0.05) (Figure 2B).

Under constant light (the L/L regime), non-significant differences were found in the mean number of matings at each time interval (range: 0.50–0.75 matings) (Tukey’s test; *p* > 0.05), meaning that there was no observable mating rhythm in these constant conditions (Figure 2C).

### 3.2. The Spatial Pattern of Mating in R. prolixus

**Figure 3:**
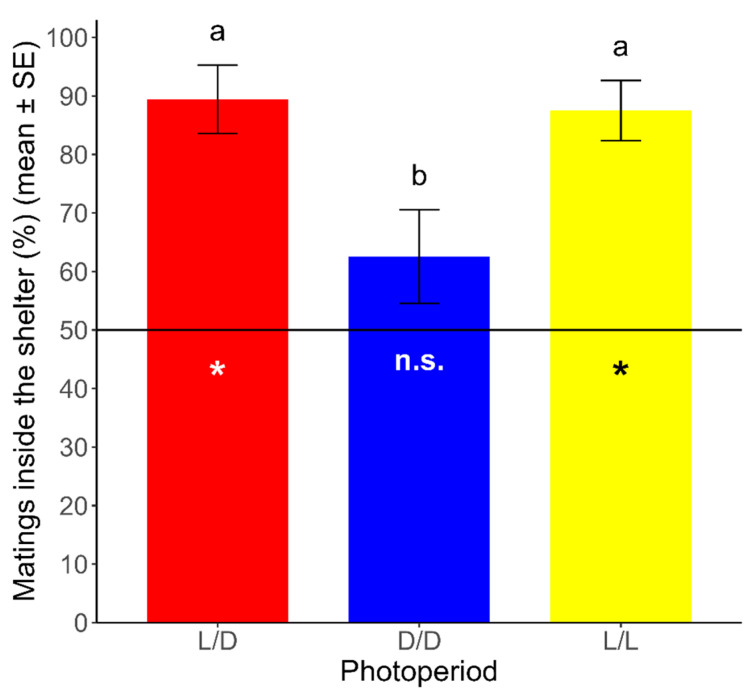
Proportion of mating inside the shelter over total mating (mean ± SE) for each photoperiod. The hatched line (y = 50) represents the expected point for a random distribution in this binary variable (inside/outside). Asterisks represent significant differences from the expected distribution (*p* < 0.05). n.s.: non-significant differences from the expected distribution (*p* > 0.05). Different lowercase letters represent significant differences in the proportions obtained between photoperiods (*p* < 0.05). (*n* = 24 for each photoperiod).

The proportion of mating inside the shelter was significantly different from a random distribution under the L/D and L/L regimes. Under the L/D and L/L regimes, 89.44 ± 5.84% and 87.50 ± 5.14% (mean ± SE) of mating, respectively, occurred inside the shelter (L/D: t = 6.752, df = 23, *p* < 0.05; L/L: t = 7.296, df = 23, *p* < 0.05). On the other hand, the proportion of mating inside the shelter was not significantly different from a random distribution under the D/D regime, where 62.57 ± 7.99% (mean ± SE) of the mating occurred inside the shelter (t = 1.573, df = 23, *p* > 0.05). Additionally, we analyzed the differences between photoperiods in the spatial pattern of mating in *R. prolixus*, using a GLM with the photoperiod (L/D, D/D, and L/L) as the explanatory variable and the proportion of mating inside the shelter as the dependent variable. We found significant differences for the proportion of mating inside the shelter between photoperiods (χ^2^ = 21.702, df = 2, *p* < 0.05), which was lower under the D/D regime than that under the L/D and L/L regimes (Tukey’s test; *p* < 0.05). Non-significant differences were found for the proportion of mating inside the shelter between both of the latter regimes (Tukey’s test; *p* > 0.05) (Figure 3).

### 3.3. The Total Number of Matings and the Distribution of Mating Across Days for Each Photoperiod

**Figure 4:**
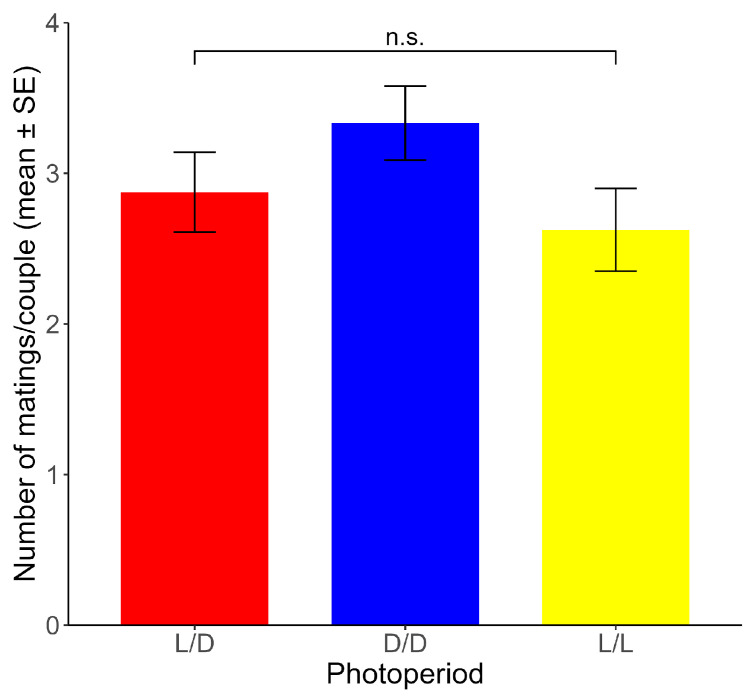
Number of matings per couple (mean ± SE) for each photoperiod. n.s.: non-significant differences (*p* > 0.05). (*n* = 24 for each photoperiod).

Non-significant differences between photoperiods were found for the mean number of matings per couple across the ten experimental days (range: 2.63–3.33 matings) (χ^2^ = 2.085, df = 2, *p* > 0.05) (Figure 4).

**Figure 5:**
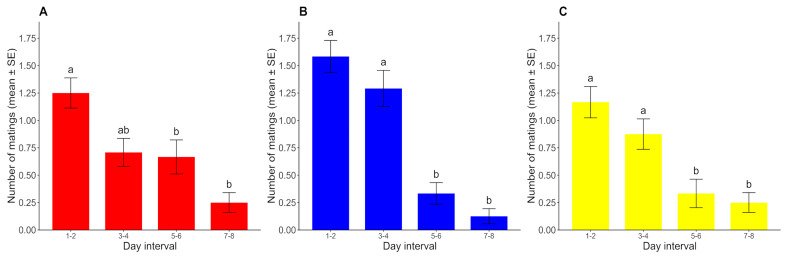
Number of matings for each day interval (mean ± SE) under (**A**) L/D, (**B**) D/D, and (**C**) L/L regimes. For each photoperiod, different lowercase letters represent significant differences between time intervals (*p* < 0.05) (*n* = 24 for each photoperiod).

Since no mating was registered after the 9th day of the assay, the 9th–10th-day interval was discarded from the analysis. The number of matings showed a decreasing trend over time for all photoperiods, with slight differences in the rate at which sexual activity declined across photoperiods. For the photoperiods with constant conditions (D/D and L/L), the number of matings remained high until the fourth day and then declined abruptly, but this decline was a little more gradual under the L/D cycle. In all three cases, the number of matings registered in days 1–2 was significantly higher than that in days 5–6 and 7–8 (*p* < 0.05) but was not significantly higher than that in days 3–4 (*p* > 0.05). Under constant conditions, the number of matings in days 3–4 was significantly higher than that in days 5–6 and 7–8 (*p* < 0.05), but these comparisons did not show significant differences under the L/D cycle (*p* > 0.05). The numbers of matings in days 5–6 and 7–8 were not significantly different from each other in any case (*p* > 0.05) (Figure 5).

## 4. Discussion

Our results show that *R. prolixus* presents a mating rhythm under a normal light:dark cycle, engaging in this behavior mainly during daylight hours. Based on the previously reported rhythms for triatomines and this new evidence, we conclude that the mating time of these insects coincides with their periods of highest aggregation rather than with their periods of highest locomotor activity, as we initially assumed [16]. This rhythm may be under endogenous control, as it persists under constant dark conditions, although the peak of mating extends into the first half of the subjective night. However, no rhythmic pattern was observed under constant light conditions. A similar situation was observed in the rhythmic pattern of the responsiveness to CO_2_ of *T. infestans* nymphs when they were exposed to constant light or dark conditions [14]. This result can not be attributed to a decrease in sexual activity, as the number of matings was not significantly different accross the photoperiods. Rhythms in nocturnal animals do not always persist under constant light, so it is thought that these conditions can interfere with pacemaker functioning in some of these species [4,43,44]. However, as long as a rhythm persists under constant darkness, the existence of endogenous control of that rhythm can still be considered, and this could be the case for the mating rhythm in *R. prolixus*. These results also show the importance of shelter in the sexual behavior of *R. prolixus*, as most of the mating happened inside the artificial shelter under the L/D and L/L cycles. On the other hand, the mating location was random under the D/D cycle. We suggest that *R. prolixus* seeks to avoid being detected through visual cues during mating. Additionally, we found a general decrease in sexual activity as the experiment period progressed, as we expected. Also, the number of matings drops more sharply after the fourth day of the assay in the photoperiods with constant conditions than that under an L/D cycle.

Initially, we did not expect to find a peak in the mating rhythm during the photophase. Many previous works have described that triatomines exhibit little to no locomotor activity across different instars during daylight hours, as their activities are primarily nocturnal [11,12,13]. Additionally, *R. prolixus* females release more volatile compounds from their metasternal glands during the initial hours of the scotophase [30]. The metasternal glands are important for the sexual behavior and communication of triatomines, triggering copulation in *R. prolixus*, *T. infestans,* and *T. dimidiata* [30,45,46]; the initiation of flight in *R. prolixus* males [47]; and the aggregation of males around mating pairs in *T. infestans* and *R. prolixus* [45,48]. The emissions of the metasternal glands during the initial scotophase suggest that *R. prolixus* males actively search for females, moving at distance, and it appears logical to conclude that reproductive behavior would also occur at night. However, previous studies have investigated the timing of behaviors and processes that are thought to be related to mating in *R. prolixus*, but none of them have specifically focused on the temporal pattern of mating. Moreover, the experimental conditions of previous works differ from those in the present study. The adult insects used by Pontes et al. [30] and Zacharias et al. [47] had no access to an individual of the opposite sex during the experiment, while the bugs used in this work had a permanently available sexual partner throughout the experiment. It is plausible that in the absence of a potential sexual partner and following a considerable amount of time without mating, *R. prolixus* adults would modify their behavior to increase the chances of encountering conspecifics of the opposite sex. Further research will be required to investigate how *R. prolixus* adults behave in such circumstances.

Hematophagy is a risky activity that requires animals dependent on access to a blood source for their survival to remain alert, as the same hosts that they bite for feeding could kill them for predation or in self-defense [49]. Additionally, copulation could be dangerous for *R. prolixus* since it lasts about 50 min, during which time both individuals remain almost stationary [25,26]. In this situation, the ability of triatomines to escape possible threats would be severely hindered, making them extremely vulnerable to predators or defensive actions by hosts. Consequently, it seems unlikely that *R. prolixus* individuals would simultaneously search for mates and hosts. A comparison between our findings and works conducted in other hematophagous insects shows different levels of association between mating and feeding rhythms. Reis and Miller [50] found that the bed bug *Cimex lectularius* feeds at night and begins to aggregate in shelter approximately two hours before the onset of the photophase. The authors proposed the search for mates as a possible explanation for this aggregation behavior. These behaviors make *C. lectularius* a species with a similar lifestyle to that of *R. prolixus*, so it is possible that feeding and mating would have divided periods in both species. Additionally, different feeding and mating rhythms and strategies have been described in hematophagous dipterans [51]. The mosquito *Anopheles gambiae* provides an example of temporal separation between mating and feeding, as it forms mating swarms at dusk and females feed on blood during the night [52,53]. Previous works suggest that a similar pattern exists in mosquito species of the *Culex pipiens* complex [54,55]. In contrast, the host-seeking and mating behaviors of the diurnal mosquito *Aedes aegypti* are less differentiated across time and space, with mating swarms occurring near hosts and in response to host odors [56,57]. Furthermore, even though male *Ae. aegypti* do not feed on blood, they are also attracted to host odors. This behavior is thought to be an integral part of their mate-searching strategy [58]. These differences in mosquito mating strategies could be attributed to the life history characteristics of these species or changes in the ecological variables of their environments, as host-based swarming is more common in species without synchronized emergence, and hosts can act as a reliable place to encounter potential mates [51]. Sandfly species of the *Lutzomyia longipalpis* complex provide another example of mating and feeding happening at similar times. These species exhibit patterns of high activity during dusk [59,60]. Males are also attracted to hosts, not to feed but to find mates, and they form leks [i.e., male display aggregations that females visit to engage in mating] near hosts during dusk; females approach them both to feed and mate [61]. This system not only may have evolved as a result of strong interaction with vertebrate hosts but also confers an important advantage for females, who gain access to a sexual partner and resources for their offspring in the same place [51,61]. As mating and feeding rhythms are clearly separated in *R. prolixus*, we suggest that its physiological limitations and gregarious behavior may have favored this strategy above others that are present in other species.

Our findings show that shelters play a crucial role in the mating behavior of *R. prolixus*, as most copulation occurs within these structures in environments with either periodical or permanent illumination. The use of refuge sites for mating has been documented in other insects. For instance, weta mate in tree cavities, commonly referred to as galleries [62]. Similarly, plant hosts provide both food and refuge for various phytophagous insects, serving as the optimal locations to find potential mates [63]. In *R. prolixus*, shelters may serve a similar function, acting as meeting points for mating pairs. In contrast, under a D/D cycle, no discernible spatial pattern was observed. These findings suggest that the negative phototaxis exhibited by *R. prolixus* plays a more significant role in their sexual behavior than their thigmotaxis. Given the lengthy duration of mating in this species, it is reasonable to think that narrow, dark places could promote mating but also that they are less inclined to seek these kinds of places in totally dark environments, as darkness may supply sufficient protection from potential dangers. We hypothesize that in wild *R. prolixus* populations under a normal light:dark cycle, encountering a potential partner in a shelter during daylight hours could be more probable and safer than doing so at the exterior, making shelters a good place for mating.

## 5. Conclusions

The results of this experiment demonstrated the existence of an endogenously controlled mating rhythm in *R. prolixus*. The use of shelters is important for the sexual behavior of these species, likely due to how bugs can interact with each other more safely inside them. The gathered evidence suggests that triatomines do not remain completely immobile during daylight hours; instead, they move around in their shelter to mate when adults of the opposite sex are present. This temporal organization would allow them to mate and feed at different hours, which could be part of a strategy to carry out these processes in a safer way, similar to that which happens in other hematophagous insects.

## Data Availability

The data are available from the corresponding author upon reasonable request.
